# Supplementary figures and images for: Long-term hematopoietic transfer of the anti-cancer and lifespan-extending capabilities of a genetically engineered blood system by transplantation of bone marrow mononuclear cells
Source: eLife. 2024 May 16;12:RP88275. doi: 10.7554/eLife.88275 (PMC11098557; doi:10.7554/eLife.88275)

Figure 4-figure supplement 2A

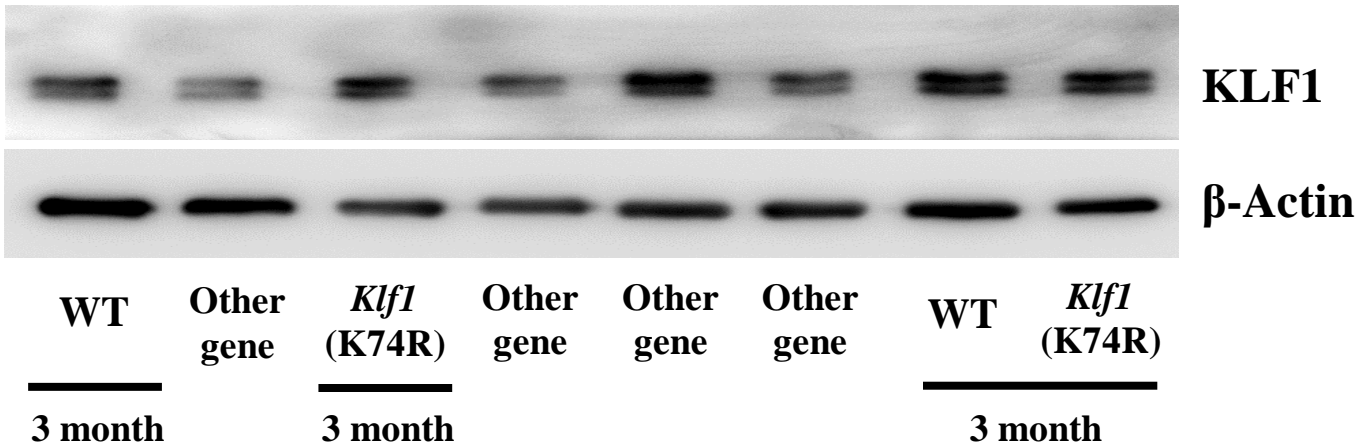

**Figure 4-figure supplement 2A**

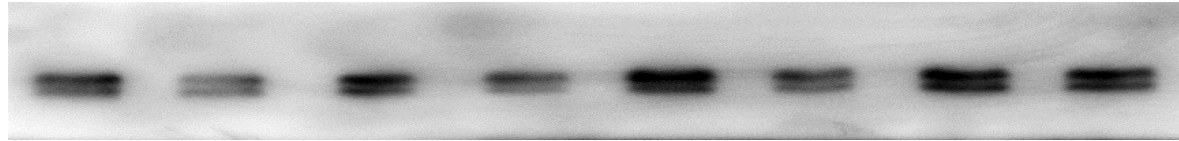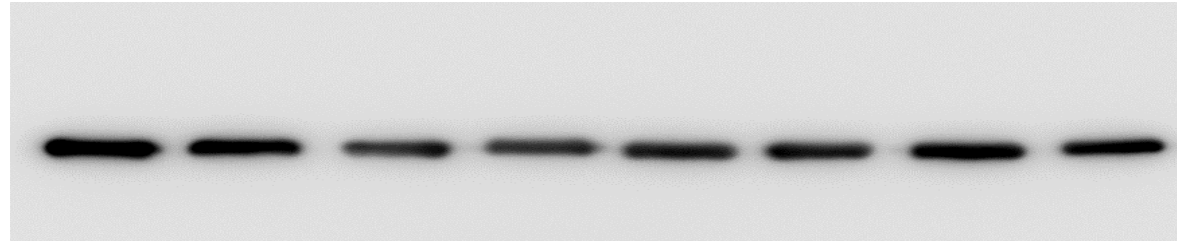

Supplement: Figure 4—figure supplement 2—source data 1. [file elife-88275-fig4-figsupp2-data1.pdf]
