# Supplementary material for: Long-term hematopoietic transfer of the anti-cancer and lifespan-extending capabilities of a genetically engineered blood system by transplantation of bone marrow mononuclear cells
Source: eLife. 2024 May 16;12:RP88275. doi: 10.7554/eLife.88275 (PMC11098557; doi:10.7554/eLife.88275)

Figure 4-figure supplement 2B

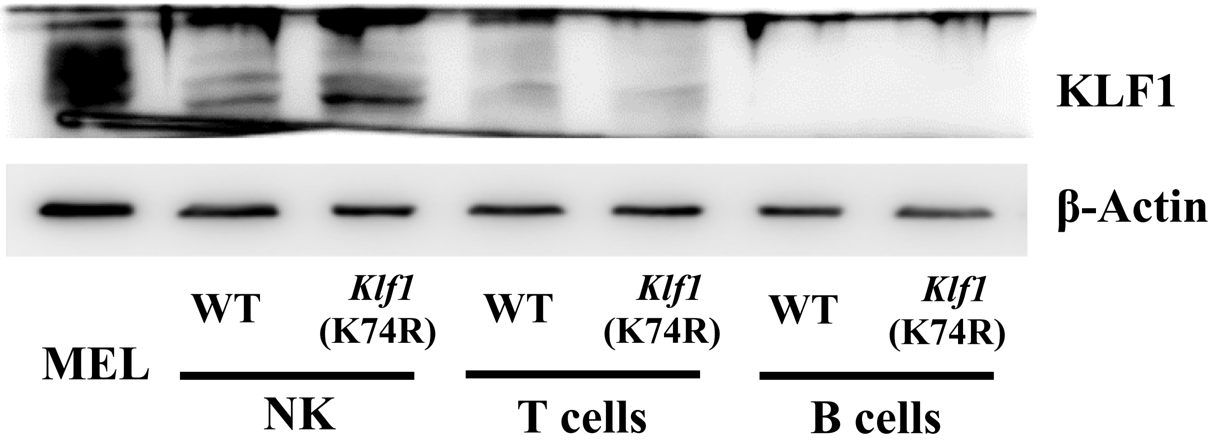

Figure 4-figure supplement 2B

KLF-1

Bright Field

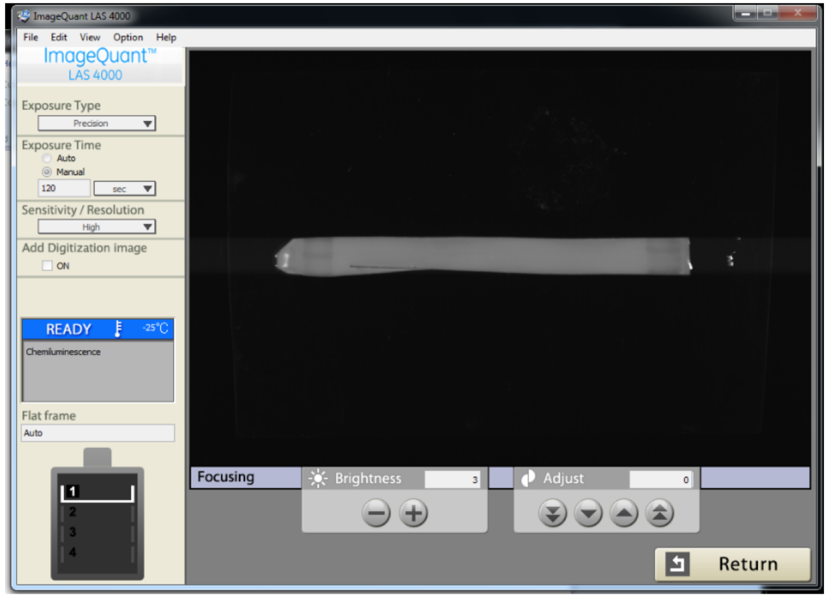

WB

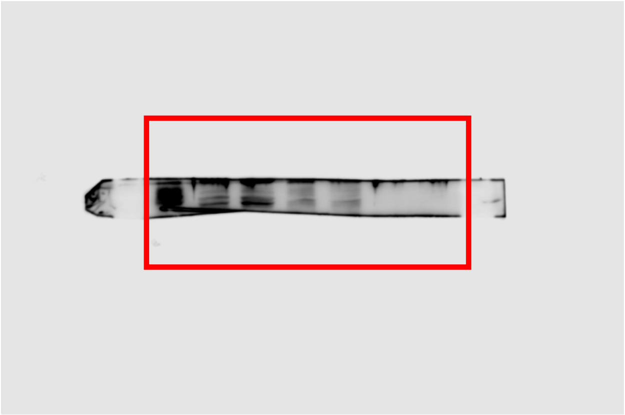

MEL WT *Klf1* (K74R) WT *Klf1* (K74R) WT *Klf1* (K74R)

NK T cells B cells

β-Actin

Bright Field

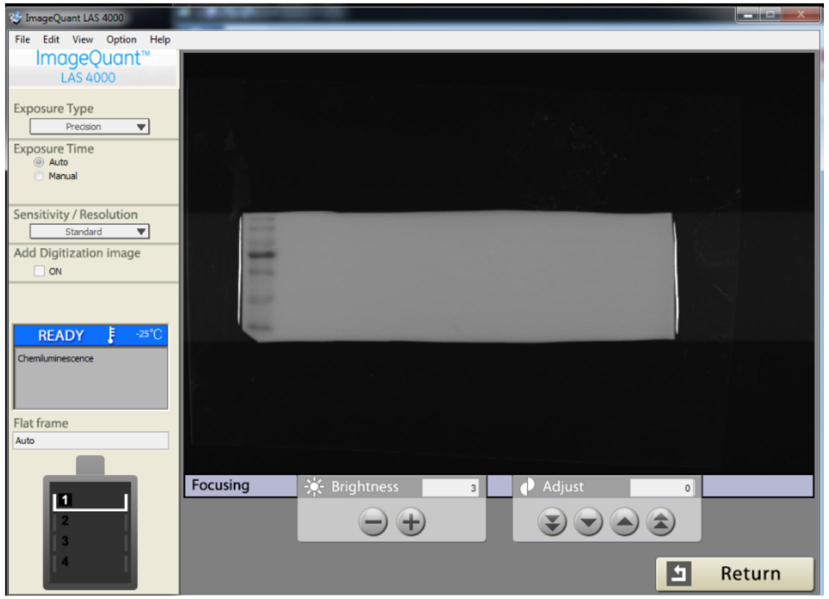

WB

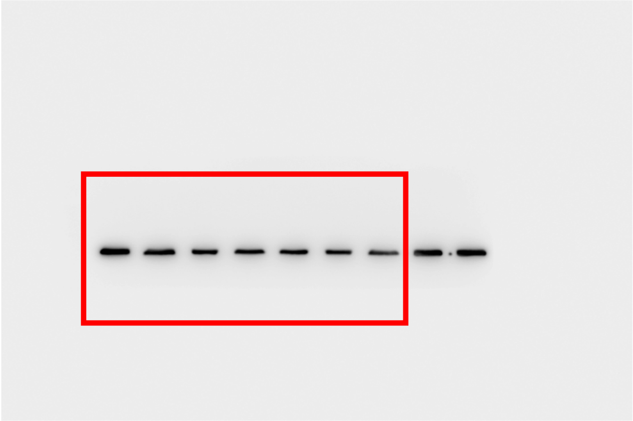

MEL WT *Klf1* (K74R) WT *Klf1* (K74R) WT *Klf1* (K74R) Other gene Other gene

NK T cells B cells

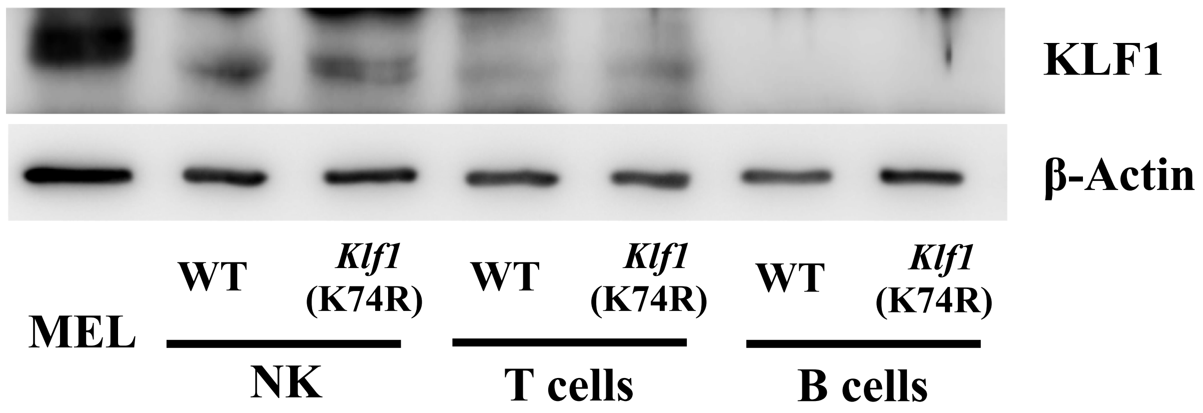
KLF1
β-Actin
MEL
WT
Klf1 (K74R)
WT
Klf1 (K74R)
WT
Klf1 (K74R)
NK
T cells
B cells

Bright Field **KLF-1** WB

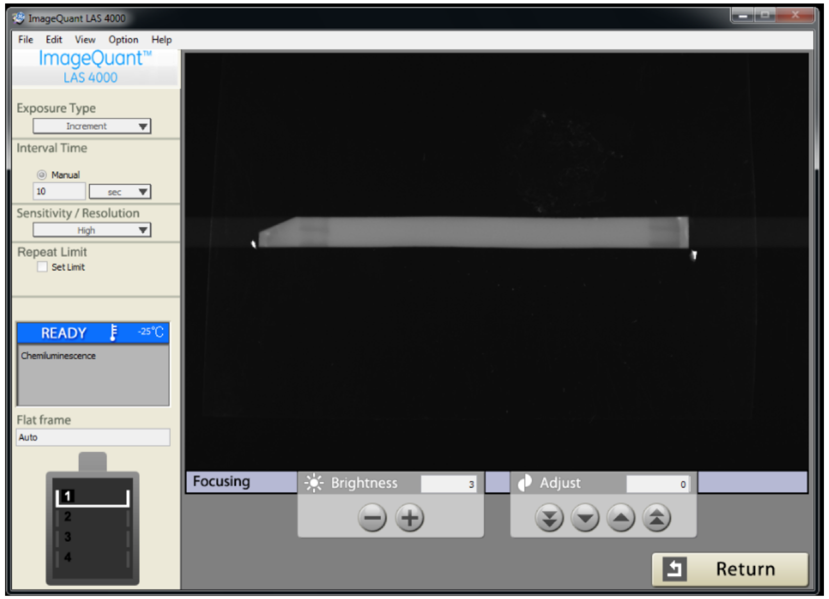

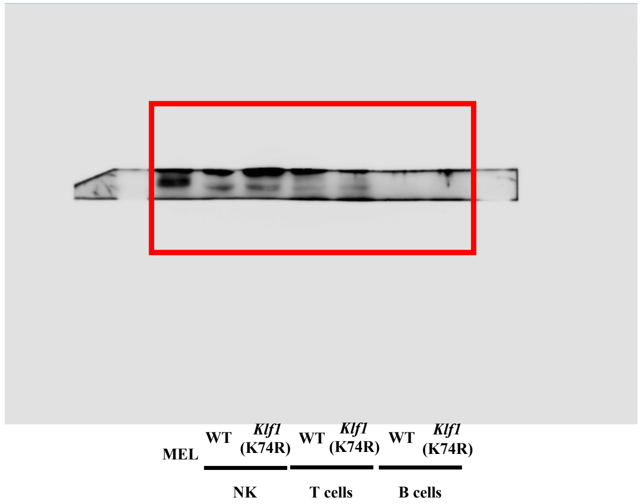

Bright Field **β-Actin** WB

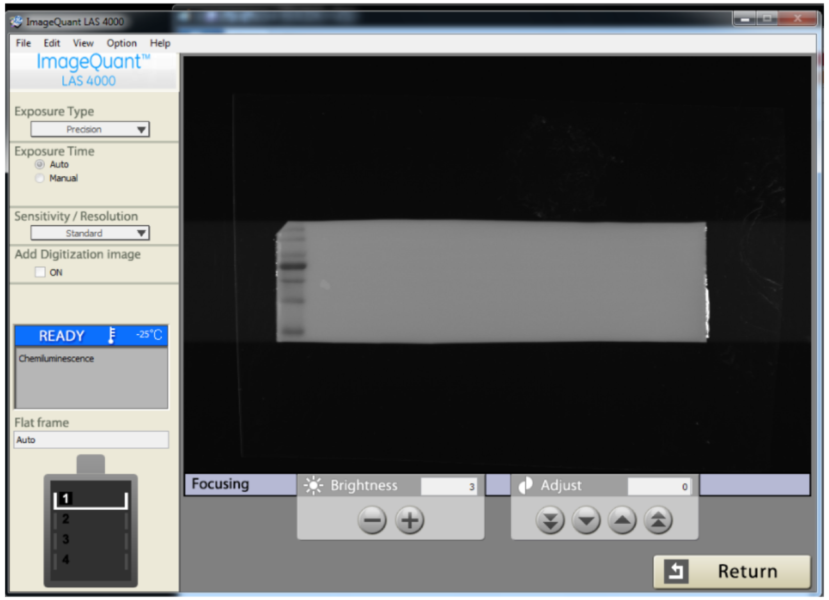

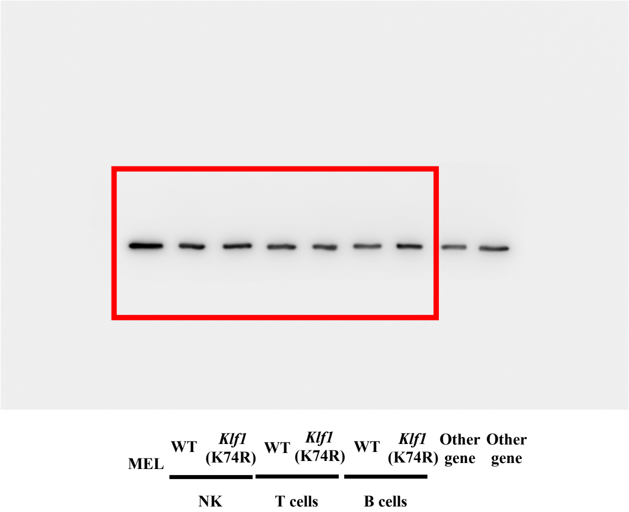

Supplement: Figure 4—figure supplement 2—source data 2. [file elife-88275-fig4-figsupp2-data2.pdf]
