# Supplementary material for: Long-term hematopoietic transfer of the anti-cancer and lifespan-extending capabilities of a genetically engineered blood system by transplantation of bone marrow mononuclear cells
Source: eLife. 2024 May 16;12:RP88275. doi: 10.7554/eLife.88275 (PMC11098557; doi:10.7554/eLife.88275)
Supplement: MDAR checklist [file elife-88275-mdarchecklist1.docx]

**Materials Design Analysis Reporting (MDAR)**

**Checklist for Authors**

The [MDAR framework](https://osf.io/xfpn4/) establishes a minimum set of requirements in transparent reporting mainly applicable to studies in the life sciences.

*eLife* asks authors to **provide detailed information within their article** to facilitate the interpretation and replication of their work. Authors can also upload supporting materials to comply with relevant reporting guidelines for health-related research (see [EQUATOR Network](http://www.equator-network.org/%20)), life science research (see the [BioSharing Information Resource](http://biosharing.org/)), or animal research (see the [ARRIVE Guidelines](http://www.plosbiology.org/article/info:doi/10.1371/journal.pbio.1000412) and the [STRANGE Framework](https://doi.org/10.1038/d41586-020-01751-5); for details, see *eLife*’s [Journal Policies](https://reviewer.elifesciences.org/author-guide/journal-policies)). Where applicable, authors should refer to any relevant reporting standards materials in this form.

For all that apply, please note **where in the article** the information is provided. Please note that we also collect information about data availability and ethics in the submission form.

**Materials:**

| **Newly created materials** | **Indicate where provided: section/figure legend** | **N/A** |
| --- | --- | --- |
| The B6 *Eklf*(K74R) mice  The FVB *Eklf*(K74R) mice  The B6 *Eklf*(K74A) mice  DOI: 10.1002/advs.202201409 | All Figures  Figure 1 (C)  Figure 1 (D) |  |
|  |  |  |
| **Antibodies** | **Indicate where provided: section/figure legend** | **N/A** |
| Anti-CD45.1 (eBiosciences Cat# 17-0453-82)  Anti-CD45.2 (eBiosciences Cat# 56-0454-82)  Anti-CD3e (eBiosciences Cat# 11-0032-82)  Anti-CD45R (eBiosciences Cat# 11-0452-82)  Anti-NK1.1 (eBiosciences Cat# 17-5941-82)  Anti-NKp46 (eBiosciences Cat# 25-3351-82)  Anti-Gr-1 (eBiosciences Cat# 11-5931-82)  Anti-CD11b (eBiosciences Cat# 12-0112-82)  Anti-PD-1 (eBiosciences Cat# 11-9985-82)  Anti-PD-L1 (eBiosciences Cat# 25-5982-82) | Figure 2 (B)  Figure 2 (B)  Figure 4 (B)  Figure 4 -Figure supplement 3  Figure 4- Figure supplement 4 (B)  Figure 4- Figure supplement 4 (C)  Figure 4 (B)  Figure 4- Figure supplement 3  Figure 4- Figure supplement 4 (B)  Figure 4- Figure supplement 4 (C)  Figure 4- Figure supplement 3  Figure 4- Figure supplement 4 (B)  Figure 4- Figure supplement 4 (B)  Figure 4- Figure supplement 3  Figure 4- Figure supplement 3  Figure 4 (A)  Figure 4 (B)  Figure 4 (C)  Figure 4- Figure supplement 4 (D)  Figure 4 (A)  Figure 4 (B)  Figure 4 (C)  Figure 4- Figure supplement 4 (D) |  |
|  |  |  |
| **DNA and RNA sequences** | **Indicate where provided: section/figure legend** | **N/A** |
| RT-qPCR mRNA sequences  *Eklf* (Forward: 5'-GGACACCCAGGAGGACTTC-3')  (Reverse: 5'-GGGTCCTCCGATTTCAGACTCA-3')  *Pd-1* (Forward: 5'-AACCGCACAGTCACGCTAC-3')  (Reverse: 5'-TGATCGTCAATTCCCGGAACA-3')  *Pd-l1* (Forward: 5'-TGCGGACTACAAGCGAATCACG-3')  (Reverse: 5'-CTCAGCTTCTGGATAACCCTCG-3')  CHIP-qPCR sequences  Actb+668L (5'-GACTACCTCATGAAGATCCTGAC-3')  Actb+817 (5'-CAGGGAAGAAGAGGATGCGGCC-3')  βmajor-6411 (5'-GTTCTCTGCACAGATAAGGACAAAC-3')  βmajor-6623 (5'-CTGATCCTACCTCACCTTATATGC-3')  Pd-1-d (Forward: 5'-GACCTAGAAATTGAGTCTAC-3')  (Reverse: 5'-CTCTTAAGGCTTTTCTTCCTTTC-3')  Pd-1 Box1 (Forward: 5'-GTACCAAAGCCAGGCCTCGAC-3')  (Reverse: 5'-CCTCCTGTGGGTAGGTTTGG-3')  Pd-1 Box2 (Forward: 5'-CTCCCCCACCTCTAGTTGC-3')  (Reverse: 5'-GGCAGAGTTGTCTGTAGCG-3') | Figure 4 (C)  Figure 4- Figure supplement 4 (C)  Figure 4 (B)  Figure 4 (C)  Figure 4- Figure supplement 4 (D)  Figure 4 (B)  Figure 4 (C)  Figure 4- Figure supplement 4 (D)  Figure 4- Figure supplement 5  Figure 4- Figure supplement 5  Figure 4- Figure supplement 5  Figure 4- Figure supplement 5  Figure 4- Figure supplement 5  Figure 4 Figure supplement 5  Figure 4- Figure supplement 5 |  |
|  |  |  |
| **Cell materials** | **Indicate where provided: section/figure legend** | **N/A** |
| Cell lines:  B16-F10 cells, ATCC (CRL-6475)  Hepa1-6 cells, ATCC (CRL-1830) | Figure 1 (A)  Figure 1 (B)  Figure 1 (C)  Figure 1 (D)  Figure 2 (A)  Figure 2 (B)  Figure 2 (C)  Figure 2 (D)  Figure 3 (A)  Figure 3 (B)  Figure 3 (C)  Figure 6  Figure 1- Figure supplement 1 (A)  Figure 1- Figure supplement 1 (B)  Figure 1- Figure supplement 1 (C)  Figure 2- Figure supplement 2 (A)  Figure 1 (E) |  |
| Primary cultures:  Bone marrow mononuclear cells (BMMNCs), WT or *Eklf*(K74R) C57bl/6 mice, male | Figure 2 (A)  Figure 2 (B)  Figure 2 (C)  Figure 2 (D)  Figure 3 (A)  Figure 3 (B)  Figure 3 (C)  Figure 2- Figure supplement 2 (A) |  |
|  |  |  |
| **Experimental animals** | **Indicate where provided: section/figure legend** | **N/A** |
| Laboratory animals or Model organisms:  C57bl/6 mice, male, Strain #:000664, RRID:IMSR_JAX:000664 | Figure 1 (A)  Figure 1 (B)  Figure 1 (D)  Figure 1 (E)  Figure 2 (A)  Figure 2 (B)  Figure 2 (C)  Figure 2 (D)  Figure 3 (A)  Figure 3 (B)  Figure 3 (C)  Figure 4 (A)  Figure 4 (B)  Figure 4 (C)  Figure 5 (A)  Figure 5 (B)  Figure 5 (C)  Figure 5 (D)  Figure 5 (E)  Figure 5 (F)  Figure 6  Figure 1- Figure supplement 1 (A)  Figure 1- Figure supplement 1 (B)  Figure 1- Figure supplement 1 (C)  Figure 2- Figure supplement 2 (A)  Figure 2- Figure supplement 2 (B)  Figure 4- Figure supplement 3  Figure 4- Figure supplement 4 (A)  Figure 4- Figure supplement 4 (B)  Figure 4- Figure supplement 4 (C)  Figure 4- Figure supplement 4 (D)  Figure 4- Figure supplement 5  Figure 5- Figure supplement 6 |  |
| Animal observed in or captured from the field: NO |  |  |
|  |  |  |
| **Plants and microbes** | **Indicate where provided: section/figure legend** | **N/A** |
| Plants: NO | No |  |
| Microbes: NO | No |  |
|  |  |  |
| **Human research participants** | **Indicate where provided: section/figure legend) or state if these demographics were not collected** | **N/A** |
| NO | No |  |

**Design:**

| **Study protocol** | **Indicate where provided: section/figure legend** | **N/A** |
| --- | --- | --- |
| NO | No |  |
|  |  |  |
| **Laboratory protocol** | **Indicate where provided: section/figure legend** | **N/A** |
| **Pulmonary melanoma foci assay**  DOI: 10.4049/jimmunol.1900998  **Subcutaneous cancer cell inoculation assay of Hepa1-6**  DOI: 10.18632/oncotarget.19367  **Flow cytometric analysis and cell sorting**  **RNAi knockdown of *Eklf* mRNA from T cells**  **RT-qPCR and cell treatment**  DOI: 10.3390/ijms21228448  **ChIP-qPCR assay**  DOI: 10.1038/sj.onc.1202916  **Western blotting assay (WB)**  ISBN 978-1-936113-42-2  ***In vivo* bioluminescence imaging**  **Bone marrow transplantation assay (BMT)**  DOI: 10.1182/blood-2003-12-4309  **Calcein-AM Cytotoxicity assay of NK cells**  **Bead-based multiplex assay of serum cytokines**  DOI: 10.3389/fvets.2019.00306  **Protein extraction**  **In-solution digestion**  **LC-MS/MS analysis**  **Protein identification**  **Gene Set Enrichment Analysis**  DOI: 10.1073/pnas.0506580102 | Figure 1 (A)  Figure 1 (B)  Figure 1 (C)  Figure 1 (D)  Figure 2 (C)  Figure 2 (D)  Figure 1- Figure supplement 1 (A)  Figure 1- Figure supplement 1 (B)  Figure 1- Figure supplement 1 (C)  Figure 2- Figure supplement 1 (A)  Figure 1 (E)  Figure 2 (A)  Figure 2 (B)  Figure 4 (A)  Figure 4 (B)  Figure 4 (C)  Figure 4- Figure supplement 3  Figure 4- Figure supplement 4 (B)  Figure 4- Figure supplement 4 (C)  Figure 4- Figure supplement 4 (D)  Figure 4- Figure supplement 5  Figure 4 (C)  Figure 4 (B)  Figure 4 (C)  Figure 4- Figure supplement 4 (C)  Figure 4- Figure supplement 4 (D)  Figure 4- Figure supplement 5  Figure 4- Figure supplement 4 (A)-source data 1  Figure 4- Figure supplement 4 (B)-  source data 2  Figure 3 (B)  Figure 2 (A)  Figure 2 (B)  Figure 2 (C)  Figure 2 (D)  Figure 3 (A)  Figure 3 (B)  Figure 3 (C)  Figure 2- Figure supplement 2 (B)  Figure 6  Figure 5- Figure supplement 6  Figure 5 (A)  Figure 5 (B)  Figure 5 (C)  Figure 5 (D)  Figure 5 (A)  Figure 5 (B)  Figure 5 (C)  Figure 5 (D)  Figure 5 (A)  Figure 5 (B)  Figure 5 (C)  Figure 5 (D)  Figure 5 (A)  Figure 5 (B)  Figure 5 (C)  Figure 5 (D)  Figure 5 (A)  Figure 5 (B)  Figure 5 (C)  Figure 5 (D)  Figure 5 (E)  Figure 5 (F) |  |
|  |  |  |
| **Experimental study design (statistics details) *** | | |
| **For in vivo studies: State whether and how the following have been done** | **Indicate where provided: section/figure legend. If it could have been done, but was not, write “not done”** | **N/A** |
| Sample size determination | Done, Figure 1 (E) |  |
| Randomisation | Done, Figure 1 (E) |  |
| Blinding | Done, Figure 1 (E) |  |
| Inclusion/exclusion criteria | Done, Figure 1 (E) |  |
|  |  |  |
| **Sample definition and in-laboratory replication** | **Indicate where provided: section/figure legend** | **N/A** |
| State number of times the experiment was replicated in the laboratory. | 3 times, Figure 1 (E) |  |
| Define whether data describe technical or biological replicates. | biological replicates  Figure 1 (E) |  |
|  |  |  |
| **Ethics** | **Indicate where provided: section/submission form** | **N/A** |
| Studies involving human participants: State details of authority granting ethics approval (IRB or equivalent committee(s), provide reference number for approval. | No human participants |  |
| Studies involving experimental animals: State details of authority granting ethics approval (IRB or equivalent committee(s), provide reference number for approval. | Institute of Animal Care and Use Committees, IACUC, at Academia Sinica. The IACUC numbers are 17-02-1052, 17-12-1142, 20-10-1528, BioTReC-110-M-005, and BioTReC-111-M-004, respectively. |  |
| Studies involving specimen and field samples: State if relevant permits obtained, provide details of authority approving study; if none were required, explain why. | No specimen and field samples |  |
|  |  |  |
| **Dual Use Research of Concern (DURC)** | **Indicate where provided: section/submission form** | **N/A** |
| If study is subject to dual use research of concern regulations, state the authority granting approval and reference number for the regulatory approval. | No |  |

**Analysis:**

| **Attrition** | **Indicate where provided: section/figure legend** | **N/A** |
| --- | --- | --- |
| Describe whether exclusion criteria were pre-established. Report if sample or data points were omitted from analysis. If yes, report if this was due to attrition or intentional exclusion and provide justification. | The criteria were all following the experiment standard protocol. |  |
|  |  |  |
| **Statistics** | **Indicate where provided: section/figure legend** | **N/A** |
| Describe statistical tests used and justify choice of tests. | Data were analyzed by Student's t test and presented as mean ± standard deviation (SD) or standard error of the mean (SEM) |  |
|  |  |  |
| **Data availability** | **Indicate where provided: section/submission form** | **N/A** |
| For newly created and reused datasets, the manuscript includes a data availability statement that provides details for access (or notes restrictions on access). | access |  |
| When newly created datasets are publicly available, provide accession number in repository OR DOI and licensing details where available. | https://doi.org/10.7554/eLife.88275.2 |  |
| If reused data is publicly available provide accession number in repository OR DOI, OR URL, OR citation. | https://doi.org/10.7554/eLife.88275.2 |  |
|  |  |  |
| **Code availability** | **Indicate where provided: section/figure legend** | **N/A** |
| For any computer code/software/mathematical algorithms essential for replicating the main findings of the study, whether newly generated or re-used, the manuscript includes a data availability statement that provides details for access or notes restrictions. | access |  |
| Where newly generated code is publicly available, provide accession number in repository, OR DOI OR URL and licensing details where available. State any restrictions on code availability or accessibility. | https://doi.org/10.7554/eLife.88275.2 |  |
| If reused code is publicly available provide accession number in repository OR DOI OR URL, OR citation. | https://doi.org/10.7554/eLife.88275.2 |  |

**Reporting:**

The MDAR framework recommends adoption of discipline-specific guidelines, established and endorsed through community initiatives.

| **Adherence to community standards** | **Indicate where provided: section/figure legend** | **N/A** |
| --- | --- | --- |
| State if relevant guidelines (e.g., ICMJE, MIBBI, ARRIVE, STRANGE) have been followed, and whether a checklist (e.g., CONSORT, PRISMA, ARRIVE) is provided with the manuscript. |  |  |

* We provide the following guidance regarding transparent reporting and statistics; we also refer authors to [Ten common statistical mistakes to watch out for when writing or reviewing a manuscript](https://doi.org/10.7554/eLife.48175).

**Sample-size estimation**

- You should state whether an appropriate sample size was computed when the study was being designed
- You should state the statistical method of sample size computation and any required assumptions
- If no explicit power analysis was used, you should describe how you decided what sample (replicate) size (number) to use

**Replicates**

- You should report how often each experiment was performed
- You should include a definition of biological versus technical replication
- The data obtained should be provided and sufficient information should be provided to indicate the number of independent biological and/or technical replicates
- If you encountered any outliers, you should describe how these were handled
- Criteria for exclusion/inclusion of data should be clearly stated
- High-throughput sequence data should be uploaded before submission, with a private link for reviewers provided (these are available from both GEO and ArrayExpress)

**Statistical reporting**

- Statistical analysis methods should be described and justified
- Raw data should be presented in figures whenever informative to do so (typically when N per group is less than 10)
- For each experiment, you should identify the statistical tests used, exact values of N, definitions of center, methods of multiple test correction, and dispersion and precision measures (e.g., mean, median, SD, SEM, confidence intervals; and, for the major substantive results, a measure of effect size (e.g., Pearson's r, Cohen's d)
- Report exact p-values wherever possible alongside the summary statistics and 95% confidence intervals. These should be reported for all key questions and not only when the p-value is less than 0.05.

**Group allocation**

- Indicate how samples were allocated into experimental groups (in the case of clinical studies, please specify allocation to treatment method); if randomization was used, please also state if restricted randomization was applied
- Indicate if masking was used during group allocation, data collection and/or data analysis
